# Surface Functionalization of Polyetheretherketone With Immobilized γ‐Polyglutamic Acid for Enhanced Apatite Formation

**DOI:** 10.1002/open.70189

**Published:** 2026-03-26

**Authors:** Toshiki Miyazaki, Yuta Sugino, Minaru Tokiwa, Jin Nakamura

**Affiliations:** ^1^ Graduate School of Life Science and Systems Engineering Kyushu Institute of Technology Kitakyushu Japan

**Keywords:** γ‐polyglutamic acid, apatite, hydrophilic treatment, polyetheretherketone, simulated body fluid

## Abstract

Polyetheretherketone (PEEK) is a lightweight, chemically stable, thermoplastic polymer with excellent strength and wear resistance. Therefore, it is used clinically as a restorative material for the spine, skull, and dental crowns. However, PEEK has the drawback of not directly bonding to bone in vivo. Previously reported surface modifications for bone bonding have mostly used inorganic materials, with few examples using organic materials. In this study, we attempted to impart bone‐bonding properties by immobilizing γ‐polyglutamic acid (γ‐PGA) on the surface of PEEK via a solution process. When UV irradiation was used to hydrophilize PEEK, more γ‐PGA was immobilized compared with acetic acid pretreatment. Samples with γ‐PGA immobilized following UV treatment tended to exhibit enhanced apatite growth in a simulated body fluid and peeling resistance. However, further improvement of the adhesion between the apatite layer and the substrate is required.

## Graphical abstract

The authors developed chemical solution process to immobilize γ‐polyglutamic acid (γ‐PGA) onto polyetheretherketone (PEEK). Prior hydrophilic treatment using UV irradiation improved the immobilization efficiency of γ‐PGA and the apatite‐forming ability in simulated body fluid compared with acetic acid treatment. Such modified PEEK is expected to be novel bone substitute with both bone‐bonding and drug delivery.

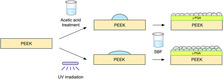

## 1 Introduction

Polyetheretherketone (PEEK) is a thermoplastic polymer with a main‐chain structure in which aromatic rings are connected by ketone and ether bonds, making it highly chemically stable and offering excellent strength, corrosion resistance, abrasion resistance, and processability. Furthermore, because it has an elastic modulus (3 to 4 GPa) close to that of human bone [1], there is low risk of stress shielding hindering the growth of surrounding bone. For these reasons, PEEK is used clinically as a spinal fixation material, such as for spinal gauges and fixation rods, as well as in cranial bone replacement and dental crown materials.

However, when PEEK is implanted into a bone defect, it is recognized as a foreign body in vivo, is isolated from surrounding tissue, and does not directly bond with living bone [2]. Various attempts have been made to address this issue. For example, a composite material has been developed by thermally mixing PEEK with bone‐bonding hydroxyapatite particles [3]. Furthermore, based on the finding that apatite formation in body fluid environments is effective in achieving bone‐binding properties [4], various surface modifications of PEEK have been attempted to induce formation of apatite with similar composition and structure to that of bone mineral. These include surface modification by concentrated sulfuric acid treatment [5, 6], surface modification with Ca^2+^ and phosphate groups [7], apatite coating by laser treatment from a supersaturated solution [8], sol–gel TiO_2_ coating [9], coating with a composite of bone‐bonding bioactive glass and chitosan [10], and surface modification with polyethylene glycol (PEG) with different terminal groups [11]. A further approach to enabling PEEK to bond with living bone, not involving apatite formation, has been to enhance osteoprogenitor cell activity by forming porous structures using 3D printing [12].

**FIGURE 1:**
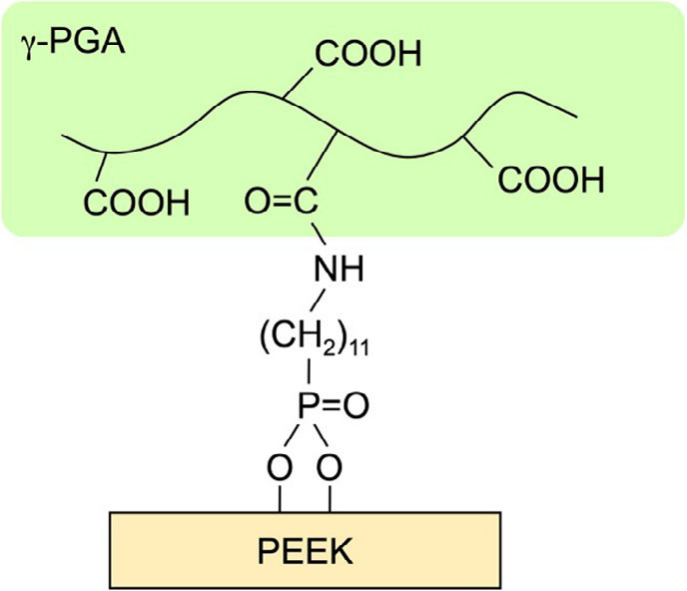
Schematic representation of polyetheretherketone (PEEK) with immobilized γ‐polyglutamic acid (γ‐PGA).

In this study, we propose a method for immobilizing γ‐polyglutamic acid (γ‐PGA) on PEEK surfaces via self‐assembled monolayers (SAMs). The predicted structure of the surface modification is shown schematically in Figure 1. γ‐PGA is a natural polypeptide derived from natto, a traditional Japanese food, and has been reported to form apatite in body fluid environments [13]. The immobilization of γ‐PGA has also been reported to impart apatite‐forming properties to Co–Cr–Mo alloys [14]. Most of the surface modifications for achieving bone bonding described above used inorganic materials, while organic modifications are relatively uncommon [10, 11]. Studies have been conducted on γ‐PGA hydrogels loaded with anticancer drugs and antibacterial Ag^+^ [15, 16]. Composites of graphene oxide, chitosan, and γ‐PGA loaded with doxorubicin enhance proliferation inhibition in Hela cells in comparison with those without γ‐PGA [17]. Also, the carboxy group of γ‐PGA can be used to control the sustained release of drugs depending on the pH environment [18]. Therefore, γ‐PGA immobilization is expected to impart both bone‐bonding abilities and pharmacological functions.

The apatite‐forming ability of PEEK substrates with immobilized γ‐PGA was evaluated in simulated body fluid (SBF) that mimics the composition of human extracellular fluid. There is a positive correlation between apatite‐forming ability in SBF and the rate of bone bonding in vivo [19]. The samples were treated with Ca^2+^ because release of Ca^2+^ enhances apatite formation by increasing the degree of supersaturation of the surrounding solution with respect to the apatite [20]. We also investigated the effects of different hydrophilic pretreatments of PEEK on γ‐PGA immobilization and apatite formation.

## 2 Experimental

### 2.1 Materials

Reagents for SBF preparation were purchased from Nacalai Tesque, Inc., Kyoto, Japan. All other reagents were purchased from FUJIFILM Wako Pure Chemical Industries, Ltd., Osaka, Japan, unless otherwise stated. The number of prepared samples was 1, unless otherwise stated.

Pure PEEK rod (PEEK450G, Yasojima Proceed Co., Ltd., Kobe, Japan), 20 cm long and 10 mm in diameter, was cut to a thickness of 1.2 mm and the surface was polished with SiC polishing paper (grain size #500). The PEEK substrate was then ultrasonically cleaned in acetone and ultrapure water for 10 min each, and dried in a drying oven (NDO‐700, Tokyo Rikakikai, Co., Ltd., Tokyo, Japan) at 60°C for 30 min.

### 2.2 Hydrophilic Treatment

For acetic acid treatment of PEEK, the PEEK plate was immersed in 5 mL of acetic acid placed in a Teflon tube at 95°C for 24 h. The specimen was then removed and dried at 60°C for 30 min. Other specimens of PEEK were irradiated with ultraviolet (UV) light for 24 h using a black light fluorescent stand (ES‐27, Sankyo Denki Co., Ltd., Kanagawa, Japan) at an irradiation distance of 50 mm. The light source was a 27 W fluorescent tube with a peak wavelength of 365 nm.

### 2.3 Immobilization of SAMs and γ‐PGA

A surface‐treated specimen was immersed in 5 mL of 1 mM 11‐aminoundecylphosphonic acid, hydrobromide (11‐AUPA; Dojindo Laboratories, Inc., Kumamoto, Japan), ethanol solution in a Teflon tube and placed at 36.5°C for 48 h. The specimen was then removed from the solution and dried at 60°C for 30 min.

**FIGURE 2:**
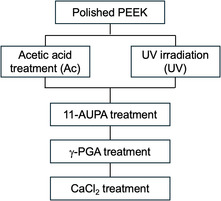
Processes of immobilization of γ‐PGA on PEEK substrates.

The specimen was then immersed in 5 mL of a mixed aqueous solution containing 15% (w/v) γ‐PGA with molecular weight of 1,000,000 (Meiji Food Materia, Co., Ltd., Tokyo, Japan), 1.8% (w/v) *N*‐hydroxysuccinimide, and 3.1% (w/v) 1‐(3‐dimethylaminopropyl)‐3‐ethylcarbodiimide hydrochloride (Tokyo Chemical Industry Co., Ltd., Tokyo, Japan) in a Teflon tube, and placed at 36.5°C for 24 h. The surface was then lightly rinsed with ultrapure water and the specimen was dried at 60°C for 30 min. The specimen was then immersed in 5 mL of 1 M CaCl_2_ aqueous solution at 36.5°C for 24 h. It was then immersed in ultrapure water for 10 min and dried at 60°C for 30 min. The series of operations is shown in Figure 2. Specimens hydrophilized with acetic acid are called Ac; those hydrophilized with acetic acid followed by γ‐PGA treatment are called Ac‐PGA; specimens hydrophilized with UV light are called UV; those hydrophilized with UV light followed by γ‐PGA treatment are called UV‐PGA.

### 2.4 SBF Immersion

Samples were soaked in 30 mL of SBF containing 142.0 mM Na^+^, 5.0 mM K^+^, 1.5 mM Mg^2+^, 2.5 mM Ca^2+^, 147.8 mM Cl^−^, 4.2 mM HCO_3_^−^, 1.0 mM HPO_4_^2−^, and 0.5 mM SO_4_^2−^ at 36.5°C for various periods up to 7 days. The solution was buffered at pH 7.40 using 50 mM tris(hydroxymethyl)aminomethane and HCl. The SBF was prepared in accordance with reported procedures [21, 22, 23]. After immersion in SBF, the sample was washed with ultrapure water for 1 h to remove salts adhering to the surface and then dried.

### 2.5 Sample Analysis

Thin‐film X‐ray diffraction (TF‐XRD; MXP3V, Mac Science Ltd., Yokohama, Japan) was used to identify the crystalline phase of the sample surface, with a CuKα X‐ray source. The voltage and current of the X‐ray tube were fixed at 40 kV and 30 mA, respectively. Step scanning mode was used with an incident angle of 1°, step angle of 0.02°, and measurement time of 1 s. Scanning electron microscopy (SEM; JCM‐7000, JEOL Ltd., Tokyo, Japan) was used to observe the sample surface. Elemental composition was analyzed by energy‐dispersive X‐ray microanalysis (EDX) combined with SEM. The chemical structures of samples were analyzed by Fourier‐transform infrared spectroscopy (FT‐IR; FT/IR‐6100, JASCO Co., Tokyo, Japan) using an attenuated total reflectance (ATR) method and X‐ray photoelectron spectroscopy (XPS; KRATOS‐AXIS‐NOVA, Shimadzu Co., Kyoto, Japan). Zinc selenide was used as a prism for FT‐IR ATR analysis. Contact angles with ultrapure water were measured using a contact angle meter (DMe‐200, Kyowa Interface Science Co., Ltd., Saitama, Japan). One‐way analysis of variance was used for statistical analysis.

### 2.6 Mechanical Properties

The surface roughness of samples was measured using a surface roughness meter (SJ‐301, Mitutoyo Co., Kawasaki, Japan) according to Japanese Industrial Standard (JIS) B 0601 [24]. Measurements were performed at 0.5 mm s^−1^ with an evaluation length of 4.0 mm. To evaluate adhesion of the formed apatite layer to the PEEK substrates, samples after immersion in SBF were subjected to a tape‐peeling test using an adhesive tape (CT‐15, NICHIBAN Co., Ltd., Tokyo, Japan) with adhesive force of 4.01 N/10 mm and a surface property measuring device (Tribogear 14DR, Shinto Scientific Co., Ltd., Tokyo, Japan) according to JIS K 5600 [25]. The feed speed of the sample stage was 6000 mm min^−1^, and the feed length was 100 mm. The surfaces after the test were observed by SEM. The area ratio of the apatite on the SEM photograph was determined by imageJ software.

## 3 Results

**FIGURE 3:**
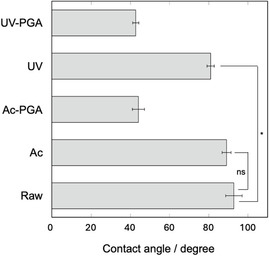
Contact angle in ultrapure water of PEEK substrates subjected to different hydrophilic pretreatments (*n* = 5). *p < 0.005, ns: no significance.

Evaluation of the contact angle is important as an index of hydrophilicity. Figure 3 shows the contact angle of each sample. The contact angle of raw PEEK was around 93°. Although the contact angle slightly decreased after acetic acid treatment, there was no significant difference compared with the untreated raw PEEK sample. However, the contact angle significantly decreased after UV irradiation. For all samples, the contact angle decreased to about 40° after treatment with γ‐PGA solution.

**FIGURE 4:**
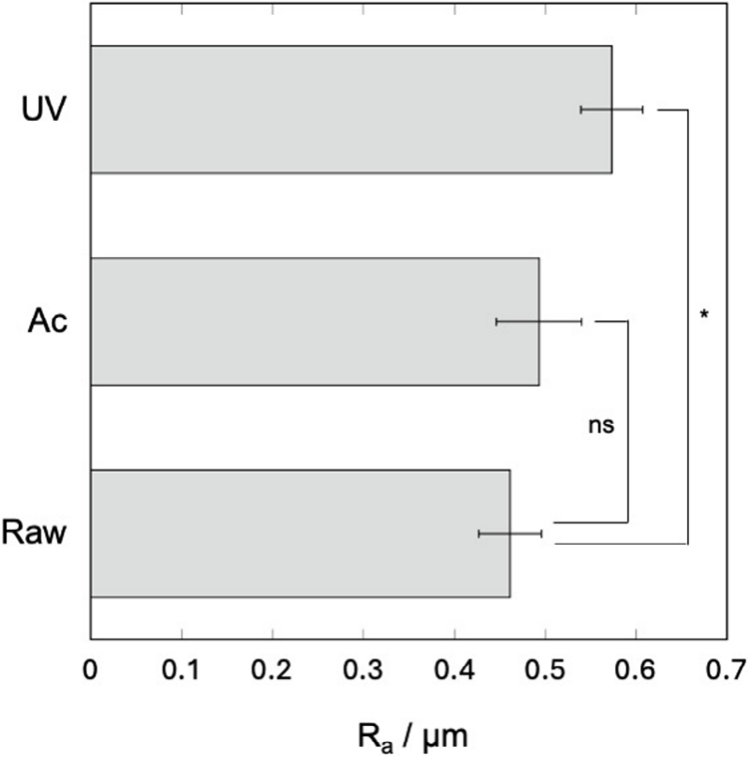
Surface roughness of PEEK substrates subjected to different hydrophilic pretreatments followed by immobilization of γ‐PGA (*n* = 10). *p < 0.005, ns: no significance.

Figure 4 shows the surface roughness of each sample. The roughness increased slightly after acetic acid treatment, but there was no significant difference compared with the untreated raw PEEK sample. There was a significant increase in surface roughness after UV irradiation.

**FIGURE 5:**
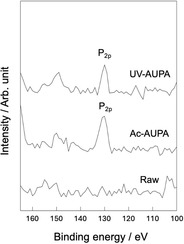
X‐ray photoelectron spectroscopy spectra of Ac‐AUPA (PEEK specimens treated with acetic acid followed by immobilization of self‐assembled monolayers) and UV‐AUPA (PEEK specimens irradiated with UV light followed by immobilization of self‐assembled monolayers).

Figure 5 shows XPS spectra of samples after hydrophilic treatment followed by immobilization of self‐assembled monolayers. P_2p_ peaks derived from 11‐AUPA were detected around 130 eV in the treated samples.

**FIGURE 6:**
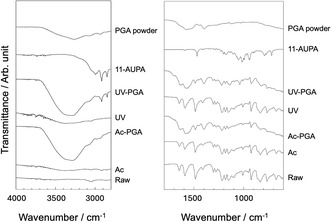
Fourier‐transform infrared attenuated total reflectance spectra of samples after hydrophilic treatment followed by immobilization of self‐assembled monolayers and γ‐PGA.

Figure 6 shows FT‐IR ATR spectra of samples after hydrophilic treatment and after hydrophilic treatment followed by immobilization of SAMs and γ‐PGA. After acetic acid treatment or UV irradiation, a hydroxyl group peak was observed near 3400 cm^−1^ in addition to the peaks derived from PEEK (shown in Table 1) [26]. After treatment with γ‐PGA solution, the hydroxyl group peak became even stronger, and peaks assigned to γ‐PGA (shown in Table 1) were also observed [27]. The γ‐PGA/PEEK ratio, calculated from the peak area derived from γ‐PGA at 1401 cm^−1^ and that derived from PEEK at 1487 cm^−1^, was slightly higher after UV irradiation (2.77) than after acetic acid treatment (2.45).

**TABLE 1:** Observed vibration modes and band frequencies in PEEK and γ‐PGA.

Compound	Wavenumber (cm^−1^)	Chemical bond
PEEK	834	C—H out‐of‐plane bending vibration in phenyl ring
	1154	C—H in‐plane bending vibration in phenyl ring
	1183, 1218	C—O stretching in diphenylether bond
	1279, 1306, 1412	C—C stretching in phenyl–carbonyl bond
	1487, 1592	Skeletal in‐plane vibration of phenyl ring
	1650	C=O stretching in ketone
	3040, 3064, 3100	C—H stretching in phenyl ring
γ‐PGA	1039	C—N stretching
	1401	C=O stretching
	1621	C=O stretching (Amide I)

**FIGURE 7:**
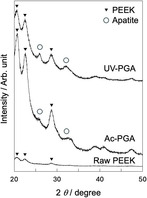
Thin‐film X‐ray diffraction patterns of Ac‐PGA (PEEK specimens treated with acetic acid followed by γ‐PGA treatment) and UV‐PGA (PEEK specimens irradiated with UV light followed by γ‐PGA treatment) after immersion in simulated body fluid (SBF) for 7 days.

**FIGURE 8:**
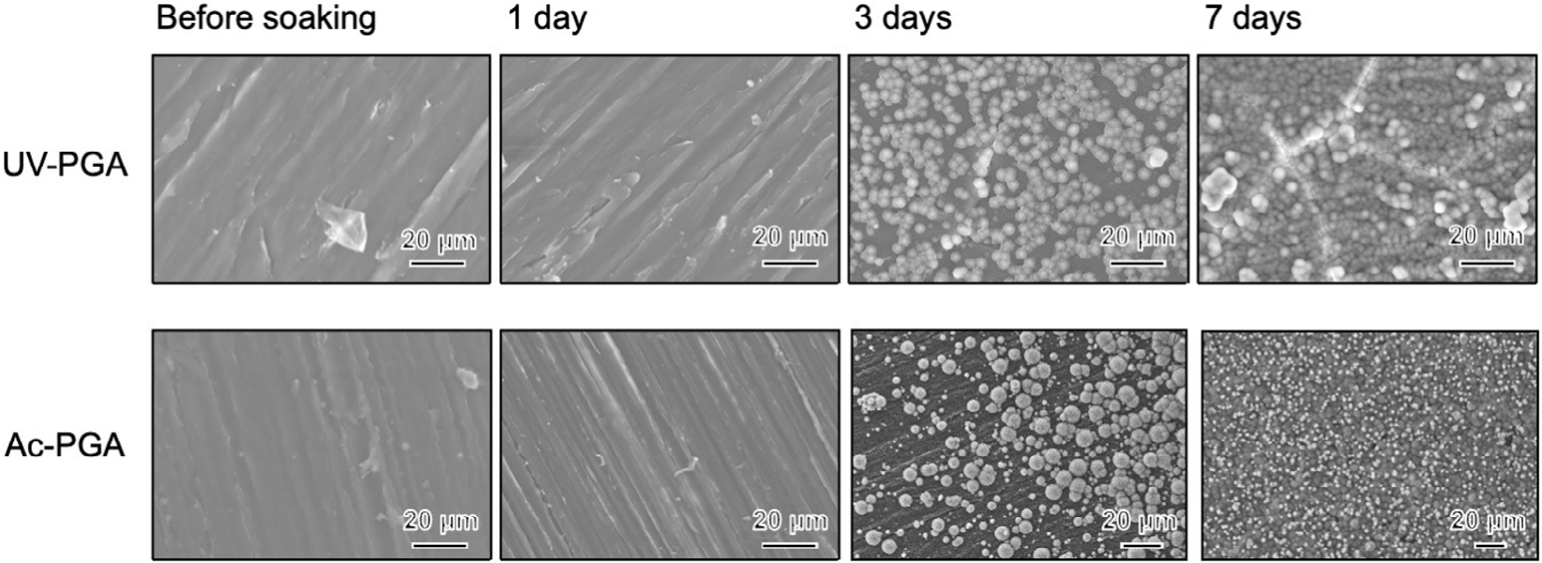
Scanning electron microscopy (SEM) images of Ac‐PGA and UV‐PGA samples after immersion in SBF for 7 days.

**FIGURE 9:**
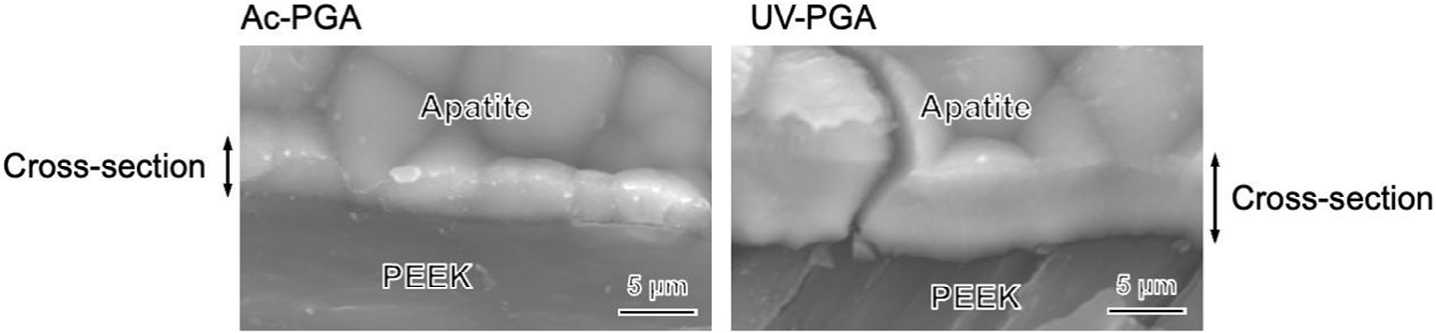
SEM images of the cross‐section of the apatite layer formed on Ac‐PGA and UV‐PGA in SBF after 7 days.

**FIGURE 10:**
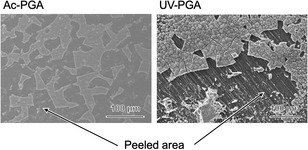
SEM images of the apatite layer formed on Ac‐PGA and UV‐PGA in SBF after the peeling‐off (tape peeling) test.

Figure 7 shows TF‐XRD patterns of samples after immersion in SBF for 7 days. Broad peaks arising from low‐crystalline apatite were observed around 26° and 32°. In SEM images (Figure 8), spherical particles began to be observed after 3 days, and they covered the whole surface of the substrate after 7 days. The Ca/P atomic ratio of the apatite formed was 1.41 for Ac‐PGA and 1.40 for UV‐PGA. Figure 9 shows cross‐sectional SEM images of the apatite layer; the thickness of the layer was ≈3 µm for Ac‐PGA and 7 µm for UV‐PGA. Figure 10 shows the results of the tape peeling test. Peeling of the apatite layer was observed for both samples. The area ratio of the apatite layer that remained after the test was 42% for Ac‐PGA and 56% for UV‐PGA, respectively.

## 4 Discussion

It was found that γ‐PGA can be immobilized on the surface of PEEK through the formation of SAMs with the amino group in the side‐chain of 11‐AUPA (Figure 5, 6). Surface pretreatment of the PEEK to form hydroxyl groups was effective for the immobilization. The present results support the mechanism shown in Figure 1. Phosphonic acid derivatives such as 11‐AUPA are immobilized on substrates with hydroxyl groups via dehydration condensation [28]. The γ‐PGA/PEEK ratio calculated from FT‐IR ATR spectra suggests that UV‐PGA immobilized slightly more γ‐PGA than Ac‐PGA. It is assumed that the SAM density is increased by the higher hydrophilicity and surface roughness following UV treatment of PEEK, resulting in the increased amount of immobilized γ‐PGA. It is thought that the UV‐treated sample had higher roughness because the ability of UV light to break the molecular chains of PEEK was greater than that of the acetic acid treatment. It has been confirmed by XPS that UV irradiation of PEEK breaks C—C and C—H bonds and newly forms O—C=O bond [29].

The PEEK substrates with immobilized γ‐PGA followed by CaCl_2_ treatment formed apatite in SBF (Figure 7, 8). It was a calcium‐deficient type, commonly seen for apatite formed in SBF [30]. Judging from SEM images, there was no significant difference in the density of the apatite between Ac‐PGA and UV‐PGA specimens (Figure 8). However, the thickness of the apatite layer was higher for UV‐PGA (Figure 9). This suggests that although there was not much difference in the rate of the heterogeneous apatite nucleation, nucleus growth was enhanced for UV‐PGA. γ‐PGA has a high ability to chelate Ca^2+^ [31]. Therefore, more Ca^2+^ would be incorporated into UV‐PGA and released into the surrounding fluid, leading to enhanced apatite formation compared with Ac‐PGA. Furthermore, apatite formation is affected by the roughness of the substrate [32]; apatite growth would be promoted on the rougher PEEK surface produced by UV irradiation.

In the case of Co–Cr–Mo alloy immobilized with γ‐PGA, apatite covered the whole substrate surface after 3 days of immersion in SBF [14]. Therefore, the apatite‐forming ability of surface‐modified PEEK is lower than that of the Co–Cr–Mo alloy. The contact angle of PEEK after hydrophilic pretreatment (>80°) was higher than that of bare Co–Cr–Mo alloy (73°). Therefore, the immobilization efficiency of γ‐PGA on PEEK is lower compared with the Co–Cr–Mo alloy because there are fewer hydroxyl groups on the PEEK surface as sites for SAM formation. This is also likely to be the cause of the peeling of the apatite layer in the tape peeling test (Figure 10). Future work will require the development of more hydrophilic PEEK, such as by shortening the irradiation distance to increase the UV intensity on the sample, and using oxygen plasma that is effective for hydrophilization [9, 33].

The molecular structure of γ‐PGA, such as its molecular weight, three‐dimensional conformation, and the ionic nature of the polymer, is also thought to affect the immobilization efficiency. These factors interact in complex ways in solution systems. Wolny et al. investigated the binding behavior of hyaluronic acid to a lipid membrane modified with a hyaluronic acid‐binding domain, and reported that as the molecular weight increased so did the immobilization density [34]. However, when immobilizing PEG on a gold surface via SAMs, high molecular weight resulted in a decrease in immobilization density [35]. In terms of the ionic nature of the polymer, a study examining the adsorption behavior of ionic poly(diallyldimethylammonium chloride) on silica glass surfaces showed that the polymer chains form a loop‐like conformation and adsorb along the substrate surface at high molecular weight [36]. If the binding mechanism of γ‐PGA with PEEK is similar to this, the carboxy group in γ‐PGA may interact only weakly with amino‐terminated SAMs through dipole–dipole interactions, potentially preventing dehydration condensation of the SAMs and γ‐PGA. In the future, it would be desirable to increase the immobilization efficiency by appropriately controlling the molecular weight of the γ‐PGA.

## 5 Conclusions

The apatite‐forming ability of PEEK with immobilized γ‐PGA was investigated in SBF. Thickness of the apatite layer in UV‐PGA group was ≈2.3 times and area ratio of the remaining apatite after the peeling test was 1.3 times compared with Ac‐PGA, indicating that UV‐based hydrophilic treatment is more effective in enhancing γ‐PGA immobilization and apatite growth. In the future, it is necessary to enhance the hydrophilic effect to improve the adhesion of the apatite layer. Furthermore, if molecular modification of γ‐PGA is used to allow sustained, rate‐optimized drug release, then the functionality of PEEK as a biomaterial is expected to improve.

## Funding

This study was supported by JSPS KAKENHI (24K01163).

## Conflicts of Interest

The authors declare no conflicts of interest.

## Data Availability

Data sharing not applicable to this article as no datasets were generated or analyzed during the current study.

## References

[1] S. Najeeb , M. S. Zafar , Z. Khurshid , and F. Siddiqui , “Applications of Polyetheretherketone (PEEK) in Oral Implantology and Prosthodontics,” Journal of Prosthodontic Research 60, no. 1 (2016): 12–19, 10.1016/j.jpor.2015.10.001. 26520679

[2] D. Almasi , N. Iqbal , M. Sadeghi , I. Sudin , M. R. Abdul Kadir , and T. Kamarul , “Preparation Methods for Improving PEEK's Bioactivity for Orthopedic and Dental Application,” A Review. International Journal of Biomaterials 2016 (2016): 8202653, 10.1155/2016/8202653. 27127513 PMC4834406

[3] S. Verma and N. Sharma , “Evaluation of Mechanical and Biotribological Performance of HAp Incorporated Polyetherketone Biocomposites for Implant Applications,” Materials Chemistry and Physics 343 (2025): 131088, 10.1016/j.matchemphys.2025.131088.

[4] T. Kokubo , H. M. Kim , and M. Kawashita , “Novel Bioactive Materials with Different Mechanical Properties,” Biomaterials 24, no. 13 (2003): 2161–2175, 10.1016/s0142-9612(03)00044-9. 12699652

[5] Y. Zhao , H. M. Wong , W. Wang , et al., “Osseointegration, and Bioactivity of Three‐Dimensional Porous and Nanostructured Network on Polyetheretherketone,” Biomaterials 34, no. 37 (2013): 9264–9277, 10.1016/j.biomaterials.2013.08.071. 24041423

[6] T. Miyazaki , C. Matsunami , and Y. Shirosaki , “Bioactive Carbon‐PEEK Composites Prepared by Chemical Surface Treatment,” Materials Science and Engineering C 70, no. 1 (2017): 71–75, 10.1016/j.msec.2016.08.058. 27770945

[7] Sunarso , A. Tsuchiya , R. Toita , K. Tsuru , and K. Ishikawa , “Enhanced Osseointegration Capability of Poly(ether ether Ketone) via Combined Phosphate and Calcium Surface‐Functionalization,” International Journal of Molecular Sciences 21, no. 1 (2020): 198, 10.3390/ijms21010198. PMC6981423 31892154

[8] A. Oyane , M. Nakamura , I. Sakamaki , Y. Shimizu , S. Miyata , and H. Miyaji , “Laser‐Assisted Wet Coating of Calcium Phosphate for Surface‐Functionalization of PEEK,” PLoS ONE 13, no. 10 (2018): e0206524, 10.1371/journal.pone.0206524. 30379904 PMC6209325

[9] T. Kizuki , T. Matsushita , and T. Kokubo , “Apatite‐Forming PEEK with TiO_2_ Surface Layer Coating,” Journal of Materials Science: Materials in Medicine 26, no. 1 (2015): 41, 10.1007/s10856-014-5359-1. 25589201

[10] K. Przykaza , M. Jurak , G. Kalisz , R. Mroczka , and A. E. Wiącek , “Characteristics of Hybrid Bioglass‐Chitosan Coatings on the Plasma Activated PEEK Polymer,” Molecules 28, no. 4 (2023): 1729, 10.3390/molecules28041729. 36838717 PMC9967460

[11] S. Zhao , W. Dong , Y. Wang , et al., “Chemical Modification Strategy to Improve Biological Activity of Carbon Fiber‐Reinforced Poly(ether ether Ketone) Implants,” ACS Applied Polymer Materials 5, no. 8 (2023): 6607–6624, 10.1021/acsapm.3c01174.

[12] H. Spece , T. Yu , A. W. Law , M. Marcolongo , and S. M. Kurtz , “S.M. 3D Printed Porous PEEK Created via Fused Filament Fabrication for Osteoconductive Orthopaedic Surfaces,” Journal of the Mechanical Behavior of Biomedical Materials 109 (2020): 103850, 10.1016/j.jmbbm.2020.103850. 32543413

[13] A. Sugino , T. Miyazaki , and C. Ohtsuki , “Apatite‐Forming Ability of Polyglutamic Acid Hydrogels in Body Environment,“ Journal of Materials Science: Materials in Medicine 19, no. 6 (2008): 2269–2274, 10.1007/s10856-007-3327-8. 18058198

[14] C. Liu , C. Matsunami , Y. Shirosaki , and T. Miyazaki , “Bioactive Co‐Cr Alloy for Biomedical Applications Prepared by Surface Modification Using Self‐Assembled Monolayers and Poly‐γ‐Glutamic Acid,” Dental Materials Journal 34, no. 5 (2015): 707–712, 10.4012/dmj.2015-064. 26438996

[15] Z. Yu , Z. Wang , Y. Chen , et al., “Programmed Surface Platform Orchestrates Anti‐Bacterial Ability and Time‐Sequential Bone Healing for Implant‐Associated Infection,” Biomaterials 313 (2025): 122772, 10.1016/j.biomaterials.2024.122772. 39190942

[16] X. Jia , Y. Wei , X. Zhang , et al., “Dual‐Drug Loaded γ‐Polyglutamic Acid Hydrogel with Hydrophilic Doxorubicin and Hydrophobic Camptothecin for Enhanced Tumor Therapy,” Chemistry‐An Asian Journal 20, no. 18 (2025): e00058, 10.1002/asia.202500058. 40625159

[17] B. Liu , C. Che , J. Liu , et al., “Fabrication and Antitumor Mechanism of a Nanoparticle Drug Delivery System: Graphene Oxide/Chitosan Oligosaccharide/γ‐Polyglutamic Acid Composites for Anticancer Drug Delivery,” ChemistrySelect 4, no. 43 (2019): 12491–12502, 10.1002/slct.201903145.

[18] H. Yuan , K. Luo , Y. Lai , et al., “A Novel Poly(L‐Glutamic Acid) Dendrimer Based Drug Delivery System with Both pH‐Sensitive and Targeting Functions,” Molecular Pharmaceutics 7, no. 4 (2010): 953–962, 10.1021/mp1000923. 20481567

[19] S. Fujibayashi , M. Neo , H. M. Kim , T. Kokubo , and T. Nakamura , “A Comparative Study between In Vivo Bone Ingrowth and In Vitro Apatite Formation on Na_2_O‐CaO‐SiO_2_ Glasses,” Biomaterials 24, no. 8 (2003): 1349–1356, 10.1016/s0142-9612(02)00511-2. 12527276

[20] C. Ohtsuki , T. Kokubo , and T. Yamamuro , “Mechanism of Apatite Formation on CaO–SiO_2_–P_2_O_5_ Glasses in a Simulated Body Fluid,” Journal of Non‐Crystalline Solids 143 (1992): 84–92, 10.1016/S0022-3093(05)80556-3.

[21] T. Kokubo , H. Kushitani , S. Sakka , T. Kitsugi , and T. Yamamuro , “Solutions Able to Reproduce In Vivo Surface Structure Changes in Bioactive Glass Ceramic A‐W,” Journal of Biomedical Materials Research 24, no. 6 (1990): 721–734, 10.1002/jbm.820240607. 2361964

[22] S. B. Cho , T. Kokubo , K. Nakanishi , et al., “Dependence of Apatite Formation on Silica Gel on Its Structure: Effect of Heat Treatment,” Journal of the American Ceramic Society 78, no. 7 (1995): 1769–1774, 10.1111/j.1151-2916.1995.tb08887.x.

[23] International Organization for Standardization (ISO) , in Implants for Surgery ‐ In Vitro Evaluation for Apatite‐Forming Ability of Implant Materials. (Geneve, 2024).

[24] Japanese Industrial Standards, JIS B 0601 , 2013.

[25] Japanese Industrial Standards, JIS K 5600 , 2014.

[26] H. X. Nguyen and H. Ishida , “Molecular Analysis of the Melting Behaviour of Poly(aryl‐ether‐ether‐Ketone,” Polymer 27, no. 9 (1986): 1400–1405, 10.1016/0032-3861(86)90041-8.

[27] A. Ogunleye , A. Bhat , V. U. Irorere , D. Hill , C. Williams , and I. Radecka , “Poly‐γ‐Glutamic Acid: Production, Properties and Applications,” Microbiology 161, no. 1 (2015): 1–17, 10.1099/mic.0.081448-0. 25288645

[28] J. Schwartz , M. J. Avaltroni , M. P. Danahy , et al., “E.S. Cell Attachment and Spreading on Metal Implant Materials,” Materials Science and Engineering: C 23, no. 3 (2003): 395–400, 10.1016/S0928-4931(02)00310-7.

[29] D. Quan , B. Deegan , L. Binsfeld , et al., “Effect of Interlaying UV‐Irradiated PEEK Fibres on the Mechanical, Impact and Fracture Response of Aerospace‐Grade Carbon Fibre/Epoxy Composites,” Composites Part B: Engineering 191 (2020): 107923, 10.1016/j.compositesb.2020.107923.

[30] H. Takadama , H. M. Kim , T. Kokubo , and T. Nakamura , “Mechanism of Biomineralization of Apatite on a Sodium Silicate Glass: TEM−EDX Study In Vitro,” Chemistry of Materials 13, no. 3 (2001): 1108–1113, 10.1021/cm0008718.

[31] T. Tsujimoto , J. Kimura , Y. Takeuchi , H. Uyama , C. Park , and M. H. Sung , “Chelation of Calcium Ions by Poly(γ‐Glutamic Acid) from Bacillus Subtilis (Chungkookjang,” Journal of Microbiology and Biotechnology 20, no. 10 (2010): 1436–1439, 10.4014/jmb.1004.04043. 21030829

[32] X. Chen , A. Nouri , Y. Li , J. Lin , P. D. Hodgson , and C. Wen , “Effect of Surface Roughness of Ti, Zr, and TiZr on Apatite Precipitation from Simulated Body Fluid,” Biotechnology and Bioengineering 101, no. 2 (2008): 378–387, 10.1002/bit.21900. 18454499

[33] J. Zhang , C. Li , G. Liu , Y. Si , J. Yu , and B. R. K. Blackman , “Effect of Low‐Pressure Plasma Treatment on the Adhesive Performance of PEEK and Co‐Polymerized PAEK Composites,” Polymer Composites 46, no. S3 (2025): S773–S786, 10.1002/pc.29997.

[34] P. M. Wolny , S. Banerji , C. Gounou , et al., “Analysis of CD44‐Hyaluronan Interactions in an Artificial Membrane System: Insights into the Distinct Binding Properties of High and Low Molecular Weight Hyaluronan,” Journal of Biological Chemistry 285, no. 39 (2010): 30170–30180, 10.1074/jbc.M110.137562. 20663884 PMC2943326

[35] Y. Teramura , K. Kuroyama , and M. Takai , “Influence of Molecular Weight of PEG Chain on Interaction between Streptavidin and Biotin–PEG‐Conjugated Phospholipids Studied with QCM‐D,” Acta Biomaterialia 30 (2016): 135–143, 10.1016/j.actbio.2015.11.003. 26546413

[36] Y. Wang and P. L. Dubin , “Capillary Modification by Noncovalent Polycation Adsorption: Effects of Polymer Molecular Weight and Adsorption Ionic Strength,” Analytical Chemistry 71, no. 16 (1999): 3463–3468, 10.1021/ac990146k.

